# Evidence-Based Approaches for Determining Effective Target Antigens to Develop Vaccines against Post-Weaning Diarrhea Caused by Enterotoxigenic *Escherichia coli* in Pigs: A Systematic Review and Network Meta-Analysis

**DOI:** 10.3390/ani12162136

**Published:** 2022-08-19

**Authors:** Eurade Ntakiyisumba, Simin Lee, Gayeon Won

**Affiliations:** College of Veterinary Medicine, Jeonbuk National University, Iksan Campus, Gobong-ro 79, Iksan 54596, Korea

**Keywords:** enterotoxigenic *Escherichia coli*, post weaning diarrhea, vaccine efficacy, target antigen, systematic review, Bayesian network meta-analysis, swine

## Abstract

**Simple Summary:**

Owing to the levels of mortality and morbidity associated with enterotoxigenic *Escherichia coli* (ETEC) inflicted diarrhea, as well as the scope of the global disease burden, several initiatives to develop effective vaccines have been launched. We conducted a meta-analysis to assess the efficacy of commercially available and candidate vaccines against ETEC-associated post-weaning diarrhea in pigs. The effectiveness of vaccines was evaluated using three clinical outcomes: mortality, diarrhea, and average daily weight gain. Subsequently, a simultaneous comparison of vaccines was conducted using a Bayesian network meta-analysis approach to generate evidence-based data on the most effective vaccine based on their target antigens. The results indicated that vaccinated pigs had a significantly lower risk of diarrhea and mortality when compared to non-vaccinated pigs. Furthermore, the findings also showed that a multivalent vaccine that targets both fimbriae and enterotoxins should be prioritized to combat post-weaning diarrhea in pigs.

**Abstract:**

In this study, we conducted a meta-analysis (MA) and systematic review to evaluate the effectiveness of vaccines against post-weaning diarrhea (PWD), caused by enterotoxigenic *Escherichia coli* (ETEC), in piglets. A Bayesian network meta-analysis (NMA) was also performed to compare the effects of combining different target antigens on vaccine efficacy. Relevant electronic databases were searched using pre-specified search terms, and 17 studies were selected based on three outcomes: diarrhea, mortality, and average daily weight gain (ADWG). In pairwise MA, the vaccinated group showed a significant decrease in diarrhea (OR = 0.124 [0.056, 0.275]) and mortality (OR = 0.273 [0.165, 0.451]), and a significant increase in ADWG (SMD = 0.699 [0.107, 1.290]) compared with those in controls. Furthermore, NMA results showed that all vaccine groups, except for group D (LT enterotoxin), were effective against PWD. Rank probabilities indicated that the F4 + F18 + LT combination was the best regimen for preventing diarrhea (SUCRA score = 0.92) and mortality (SUCRA score = 0.89). NMA also demonstrated that, among the vaccine groups, those inducing simultaneous anti-adhesion and antitoxin immunity had the highest efficacy. Our results provide evidence-based information on the efficacy of vaccines in reducing PWD incidence in pigs and may serve as guidelines for antigen selection for commercial vaccine development in the future.

## 1. Introduction

Post-weaning diarrhea (PWD) is an economically important disease that affects piglets within the first 2 or 3 weeks of weaning [1]. This disease leads to significant economic losses due to the high mortality rate, weight loss, treatment cost, and growth retardation it causes in piglets. Enterotoxigenic *Escherichia coli* (ETEC) possess fimbrial antigens (adhesins) that mediate bacterial colonization by binding to specific receptors on intestinal epithelial cells [2,3]. ETEC, which express F4 (K88) or F18 adhesive fimbriae, are considered the main causative agents for PWD [4,5,6,7]. F4 and F18 adhesins are present in various ETEC variants. F4 (K88) antigenic variants include F4ab, F4ac, and F4ad; F4ac being the most common variant [4]. F18-ETEC is classified into F18ab and F18ac, which are associated with edema and diarrhea, respectively, in weaned piglets [1]. F4/F18 fimbriae and enterotoxins are crucial virulence factors involved in disease pathogenesis. Vaccination of pregnant sows imparts passive colostral and lactogenic immunity in piglets, which helps to control neonatal diarrhea [2,8]. However, as passive lactogenic immunity is gradually lost with age and is terminated completely during the weaning period [2,9], newly weaned piglets are highly vulnerable to ETEC infections. Furthermore, several studies indicate that the swine genotype may affect the susceptibility or resistance of pigs toward ETEC infection. Single nucleotide polymorphisms (SNPs) localized on alpha (1,2)-fucosyltransferase (FUT1) and bactericidal/permeability-increasing protein (BPI) genes are responsible for swine F18-ETEC susceptibility [10,11,12], while the porcine susceptibility or resistance to F4ac-ETEC is controlled by the Mucin4 (MUC4) and Mucin13 (MUC13) genes [13,14,15]. Genetically, some piglets lack the fimbriae receptor genes, and thus may be resistant to ETEC infections [1,16]. The presence or absence of intestinal receptors for ETEC attachment and colonization determines the probability of the piglets developing PWD [8,17]. The discovery that bacterial adhesion to intestinal enterocytes is the first and most important step in the development of PWD led to the development of vaccines that primarily target ETEC adhesins to prevent bacterial colonization of the small intestines [18]. This demonstrates the significance of antigen-specific receptor genes for the effectiveness of ETEC vaccines. The existence of ETEC-receptors on intestinal epithelial cells is also essential for the induction of protective mucosal immunity following swine immunization [19]. Van den Broeck et al. (1999) also previously demonstrated that no systemic or mucosal immunity could be produced in pigs lacking F4-receptors following oral immunization with purified F4 fimbriae and subsequent challenge infection [18].

After colonization and proliferation in the pig intestines, ETEC secrete one or two types of heat-labile (LT) and heat-stable (ST) enterotoxins. The LT consists of a single A domain and five B subunits possessing 240 and 103 amino acids, respectively. The B subunits primarily bind to the GM1 ganglioside receptor, acting as cell surface receptors [20]. Once the B subunits have anchored the toxin molecule to the cell surface, a fragment of the A domain (A1) will activate the adenylate cyclase system (increasing cAMP), resulting in increased fluid and electrolyte secretion and decreased absorption [1,21]. The STa toxicity is mediated by stimulation of the guanylate cyclase system, which results in intracellular accumulation of cGMP and decreased absorption of water and electrolytes on villus tips, as well as increased secretion of chloride and water in crypt cells [6]. On the other hand, when STb binds to its receptors, Ca^2+^ enters the cells, causing duodenal and jejunal secretion of water and electrolytes [8].

In the pig industry, antimicrobials and feed additives have been widely used to prevent ETEC infections, but neither strategy has provided desirable outcomes in combating PWD [8,22,23]. With the excessive use of antibiotics, bacterial resistance has become a major health concern in both animals and human beings [17]. Alternative approaches are needed to effectively protect weaned piglets against ETEC infections. Immunization of pigs is the most reliable and effective strategy wherein vaccines induce anti-adhesion immunity to block ETEC colonization and/or antitoxin immunity to neutralize enterotoxicity [23]. Several vaccine formulations have been designed and their efficacy against PWD in weaned piglets has been assessed.

Currently, systematic review and meta-analysis (SRMA) is considered the best available knowledge resource for clinicians to make decisions regarding treatment choices [24]. Le Boedec stated that systematic reviews and meta-analyses (MA) are widely perceived as the best tools for obtaining reliable evidence on different medical questions; for example, for evaluating the treatment effect or prevalence of diseases [25]. In veterinary science, MA is gaining interest as a means of evaluating the efficacy of new technologies for regulatory purposes; for example, in assessing the efficacy and safety of treatments focusing on animal health, production, and reproduction [26]. If carefully conducted, MA can provide more robust and plausible information on a particular research topic and may also explain the sources of heterogeneity between the results of individual studies [27]. When conducting pairwise MA on the effectiveness of intervention strategies, the main purpose is to draw inferences about whether one intervention is more or less effective than another in responding to a particular disease or condition of interest. However, clinical studies containing head-to-head comparisons of various preventive approaches are sparse due to financial and logistic constraints [28], which makes it difficult to establish authentic evidence on the relative effectiveness of several treatments through a conventional pairwise MA [29].

Network MA (NMA) is a statistical approach used to analyze the relative efficacy of more than two interventions, including some that have never been compared directly in the primary literature [30,31]. The use of NMA to compare treatment efficacy yields more precise estimates, takes into account all available evidence to inform decision makers [30,31,32], and generates the ranking of treatments in terms of their relative efficacy or safety [30,33]. Due to its ability to combine both direct (i.e., evidence from direct head-to-head comparisons) and indirect (i.e., evidence from a network of interventions, which have not been compared directly in the original studies but can be compared indirectly by using a common comparator such as a placebo) evidence, NMA has been considered a useful tool for estimating the effect size among several treatment groups, even though they were not compared directly in different clinical trials [31,34]. Lee et al. (2022) used a Bayesian NMA approach to compare vaccine effectiveness in preventing swine edema caused by Shiga toxin-producing *E. Coli* and ranked the vaccines in order of their efficacy [35]. Calzetta et al. (2020) also conducted an NMA to evaluate the geographical use of anthelminthic medicinal plants in livestock in the European Union and to quantify the anthelminthic efficacy of medicinal plants in comparison with that of anthelminthic drugs [36].

In clinical studies, different vaccine types, such as live attenuated, subunit, inactivated, and recombinant toxoid vaccines, and various routes of administration have been explored to induce a rapid immune response after immunization. Although scientific evidence from individual literature supports the use of vaccines for the prevention of PWD, no SRMA of the relevant studies have been conducted to obtain a quantitative summary of the results. In this study, we performed a systematic review of the available literature on the effectiveness of ETEC vaccines in preventing PWD and used an MA to generate quantitative effect estimates. A conventional pairwise MA can indicate whether vaccines are effective; however, it cannot provide information on which treatment works best out of several different regimens. To solve this issue, a Bayesian NMA model was also applied to investigate the comparative efficacy of vaccines based on their target antigens to guide clinical decision-making and vaccine manufacturers.

## 2. Materials and Methods

A systematic review and MA were conducted in accordance with the Preferred Reporting Items for Systematic Reviews and Meta-Analyses (PRISMA) checklist [37] and its extended version incorporating NMA (PRISMA-NMA) [38] to identify data available on anti-ETEC vaccines used for preventing PWD in swine. The review protocol was not registered, but it was agreed upon by all authors prior to the start of the study.

### 2.1. Research Question and Search Strategy

The review question was formulated in accordance with the “population, intervention, comparator, and outcome” (PICO) format. In this study, the population of interest refers to the weaned piglets. The intervention was immunization of pigs to protect against PWD. The comparator was a group of pigs that did not receive any of the vaccines under study (i.e., no vaccine was given or they received a placebo). The outcomes of interest included diarrhea, mortality, and the average daily weight gain (ADWG) in piglets from both experimental groups.

An extensive literature search was performed using MEDLINE (via PubMed), CAB Abstracts, and Korean databases, such as RISS and KISS, to identify relevant studies published between 1980 and 2021. The following search terms were used: (*Escherichia coli* OR *E. coli*) AND (F4 OR K88 OR F18 OR ETEC) AND (diarrhea OR Colibacillosis) AND (immune * OR vaccin * OR interve * OR treatment OR efficacy OR effect OR protect OR shed OR coliprotec OR mitigate * OR control) AND (swine OR pig or pigs OR piglet OR piglets OR gilt OR gilts OR sow OR sows OR hog OR hogs OR weaner OR feeders OR finisher OR finishers). An asterisk was used to extend a search term to related words with the same meaning (e.g., Immun * for immunization, immunity, and immunogenicity). Eleven additional articles were obtained from the Jeonbuk National University Library/Center for Foreign Academic Support in the Field of Agriculture, Forestry, and Fisheries.

### 2.2. Eligibility and Exclusion Criteria

The inclusion criteria for this review and Bayesian NMA were as follows: primary studies (i.e., original articles, not reviews) and articles that evaluated the efficacy of vaccines against PWD in a pig model, under either natural exposure or experimental challenge. Other criteria included articles that evaluated vaccine efficacy targeting F4-ETEC, F18-ETEC, or both, as well as studies that used commercially available or experimental vaccines (vaccine candidates) and were published between 1980 and 2021 with no language restrictions. Research wherein the experiment was conducted on animals other than pigs, reviews, and in vitro studies were excluded. Studies that evaluated antibody titer response post immunization rather than clinical outcomes and articles for which the full text was not available were also excluded. Titles and abstracts were screened for suitability based on the set criteria. The full texts of potentially relevant articles were obtained and assessed for eligibility.

### 2.3. Data Extraction

Two independent reviewers performed data extraction. The following data were extracted using a predeveloped Excel spreadsheet: name of the first author, year of publication, study design, type of vaccine, vaccine antigen, vaccination time, weaning age, breed, genotype, the source of animals, challenge strain, dose of vaccine, time of challenge, total number of animals, number of animals in each group, monitoring period after challenge, and reported outcomes. From each group, the number of events for dichotomous outcomes and the mean and standard deviation (SD) for continuous outcomes were calculated. In some studies where outcomes were reported graphically, WebPlotDigitizer-Version 4.4 (Ankit Rohatgi, Pacifica, CA, USA) was applied to extract data from graphs [39]. If more than one trial was conducted in a single study (e.g., using different participants, different vaccine strains or different doses, or different challenge strains), the data from each trial were extracted separately.

### 2.4. Risk of Bias Assessment

The quality of the included studies was assessed by two independent reviewers using the Animal Research: Reporting in Vivo Experiments 2.0 (ARRIVE 2.0) checklist [40]. Eighteen risk-of-bias items were evaluated, which were judged as either low, high, or unclear risk of bias. Any disagreements were resolved by discussion among the reviewers to reach a consensus. A risk-of-bias graph was generated and presented.

### 2.5. Statistical Analyses of the Data

#### 2.5.1. Pairwise MA

Comprehensive MA (CMA) software version 3 (Biostat Inc., Englewood, NJ, USA) was used to perform a conventional pairwise MA of 17 studies, comprising 37 trials. The selected studies were categorized according to the three outcomes of interest (diarrhea, mortality, and ADWG). The results of the meta-analysis were presented in forest plots as pooled odds ratio (OR) for dichotomous outcomes and standardized mean difference (SMD) for continuous outcomes with 95% confidence intervals (CI) using a random-effects model. The heterogeneity between studies was evaluated using the Q test and *I^2^* statistic [41]. The Q-statistic provides a null hypothesis that all studies in the analysis share a common effect size. *I^2^* addresses the proportion of observed variance that reflects the variance in true effects, rather than sampling errors. A statistically significant *p*-value (*p* < 0.05) and *I*^2^ > 50% indicated substantial heterogeneity. Subgroup analysis was performed to compare effect sizes between groups and identify moderators associated with the variance in effect size. Three prespecified moderators were analyzed: vaccine antigen (one or more fimbriae, one or both enterotoxins, combined fimbriae and enterotoxins), vaccine category (commercial or experimental), and route of administration (oral, intranasal, parenteral, or combined). Publication bias was visually appraised based on the symmetry of the funnel plots. The quantitative estimate of publication bias was conducted using Egger’s regression test to address the relationship between the treatment effect and standard error due to the sample size [42]. When publication bias was confirmed, the Duval and Tweedie trim-and-fill method was applied to estimate an unbiased effect by imputing the missing studies in the funnel plot [42,43].

#### 2.5.2. Bayesian NMA

The Bayesian NMA was performed using the “gemtc” package in R software to compare the effectiveness of ETEC vaccines against PWD based on their target antigens. We also evaluated the probability that each vaccine in the network was the most efficacious regimen using the surface under the cumulative ranking (SUCRA) score [44]. A uniform distribution with an average of 0 and variance of 5 was used as the prior model (*σ*∼*U*(0, 5)). The effect size was estimated as the OR with 95% credible intervals (CrIs) for diarrhea and mortality outcomes and mean difference (MD) for daily weight gain outcome using the random effects model. The Markov Chain Monte Carlo (MCMC) simulation was adapted to generate posterior samples implemented using the Just Another Gibbs Sampler (JAGS) software version 4.3.0 and “rjags” package in R software [45]. Four chains were simulated and 100,000 iterations were performed. Of these 100,000 iterations, 10,000 ‘burn in’ iterations were discarded to exclude the effect of the initial values of the algorithm. Every 20th value was extracted, which resulted in 5000 data points in each chain. Trace plots and their corresponding density plots, the Gelman–Rubin statistics, and plots were used to ensure Bayesian model convergence [46]. Graphical representations of all treatment comparisons were generated to appraise the network geometry. The outcomes of the NMA are valid if the transitivity assumption is achieved. Transitivity assumes that all covariates or effect modifiers are equally distributed across trials within a network [30]. This assumption cannot be tested statistically; however, there are several approaches to assessing inconsistency in NMA models. Consistency is a statistical representation of transitivity and indicates whether the direct and indirect effect estimates are congruent [38,47]. The presence of systematic differences between direct and indirect treatment comparisons (inconsistency) was evaluated using the node-splitting method [48,49]. The assessment of inconsistency is executed by comparing the direct evidence between two interventions with indirect evidence calculated for that same comparison in a network [49,50]. A statistically significant *p*-value (*p* < 0.05) indicates a significant disagreement between direct and indirect evidence from treatment comparisons [49].

## 3. Results

### 3.1. Search Results

**Figure 1:**
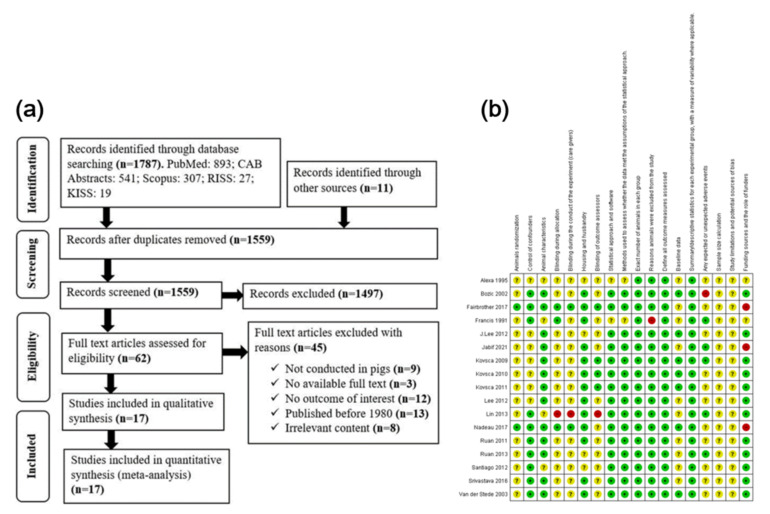
(**a**) Selection of studies used in the systematic review and network meta-analysis (PRISMA flow chart) to determine the efficacy of enterotoxigenic *Escherichia coli* (ETEC) vaccines against post-weaning diarrhea (PWD) in weaned piglets. (**b**) Risk of bias assessment of eligible studies using the ARRIVE checklist. A green circle with a plus sign (+) denotes a low risk of bias, a red circle with a negative sign (−) denotes a high risk of bias, and a yellow circle with a question mark (?) indicates an unclear risk of bias.

A total of 1798 studies were identified through electronic database searches and other sources. After removing duplicates, and evaluation of study titles and abstracts followed by full-text screening, 17 studies were found to be eligible for systematic review and NMA. The selection process and reasons for excluding studies are shown in the PRISMA flow diagram (Figure 1a).

### 3.2. Study Characteristics

The final review of the manuscripts revealed 17 studies, which included 37 trials, that were eligible for the MA. In total, 3 of the 17 studies [4,9,51], which included 9 trials, assessed the efficacy of commercially available vaccines against PWD, and the remaining studies [7,52,53,54,55,56,57,58,59,60,61,62,63,64], which included 28 trials, assessed the efficacy of experimental vaccines (candidate vaccines). Only one natural exposure field study [55] consisting of three trials, was included in the analysis, and the remaining studies assessed the effectiveness of ETEC vaccines in experimental challenge models. Studies varied highly in vaccine formulation, dosage, route of administration, frequency of immunization, and monitoring period post-challenge. Regarding vaccine delivery, four studies [54,56,58,60], which included 10 trials, used parenteral (intramuscular) vaccines, one study [61], which included three trials, used intranasal vaccines, and the remainder used oral vaccines. A summary of the included studies is presented in Table S1.

### 3.3. Quality Assessment

The risk-of-bias assessment showed that none of the studies had a low risk of bias in all risk domains (Figure 1b). No single study had reported how the sample size was obtained and they were all judged to be at unclear risk of bias for the “sample size calculation” item. Similarly, none of the studies reported limitations that could have possibly distorted their findings; therefore, they were judged to be at unclear risk of bias for “study limitations and potential sources of bias”. Only two studies [4,9] clearly described their animal randomization protocol, and they were rated as low risk for “Animals randomization.” One study [61] did not blind observations due to the limited number of investigators and was rated as high risk for “Blinding items.” All the information regarding the pig genotype, sex, breed, age, and the source of animals also was evaluated under the “Animal characteristics” and “Control of cofounders” risk of bias items. Most of the studies were judged to be at low risk of bias for both items since they clearly mentioned the ETEC-receptor status among the pigs involved in the experiments. A large number of studies included in the meta-analysis have only used receptor-positive pigs in their trials.

One study [52] had a high risk for attrition under “Reasons animals were excluded from the study” due to high losses up to follow-up. One study [54] reported that vaccinated pigs developed long-lasting diarrhea after vaccination and was rated as having a high risk of bias because the role of the challenge strain in the development of diarrhea was uncertain. Three studies [4,9,64] were judged to be at high risk for “funding sources and the role of funders in the study” due to possible conflicts of interest. Generally, most studies had a low or unclear risk of bias for all 18 risk-of-bias items adapted from the ARRIVE guidelines 2.0 checklist [40].

### 3.4. Pairwise MA Results

#### 3.4.1. Diarrhea Outcome

**Figure 2:**
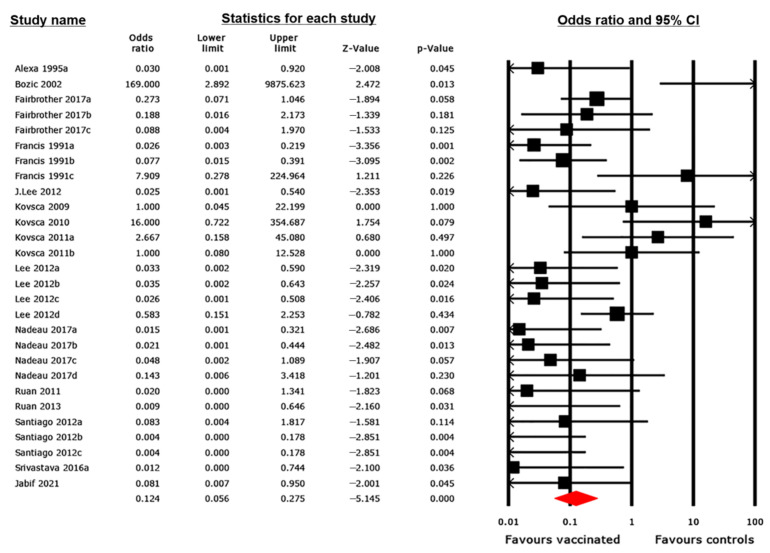
Forest plot of 28 trials assessing the effectiveness of enterotoxigenic *Escherichia coli* (ETEC) vaccines based on diarrhea outcome. The pooled effect is estimated in odds ratio (OR) with its corresponding 95% confidence interval, using a random-effects model. The black squares represent estimated ORs for each study; the size of the squares corresponds to the weight attributed to each study; horizontal solid lines represent 95% CIs; and the red diamond represents the pooled OR estimated from all studies included in the meta-analysis [4,7,9,51,52,53,54,56,57,58,59,60,62,63,64].

Fifteen studies, composed of 28 trials, assessed the efficacy of ETEC vaccines based on diarrheal outcomes (Figure 2). The pooled OR was 0.124 (95% CI 0.056, 0.275), suggesting a significantly lower risk of diarrhea in vaccinated pigs compared with that in controls (*p* < 0.001). Between-study heterogeneity was high (*I*^2^ = 57%). To investigate the source of heterogeneity, a subgroup analysis was performed based on three prespecified effect modifiers of the vaccine (antigen, route of administration, and vaccine category). A statistically significant relationship was observed between the effect size and type of antigen included in the vaccine (Table 1). The Q statistic, which tested the null hypothesis that all subgroups share a common effect, yielded a statistically significant value (Q = 19.162, *p* = 0.014). We rejected the null hypothesis and confirmed the difference in effect sizes between subgroups due to differences in the target antigens. Thus, a vaccine’s target antigen contributed to between-study heterogeneity. In contrast, when studies were grouped based on the vaccination route (oral, intranasal, parenteral, or combined) or vaccine category (commercial or experimental), no statistically significant difference was observed in effect size between the subgroups (Table S2). Thus, no relationship was confirmed between the observed effect size and route of administration (Q = 0.362, *p* = 0.834) or vaccine category (Q = 0.115, *p* = 0.734).

#### 3.4.2. Mortality Outcome

**Figure 3:**
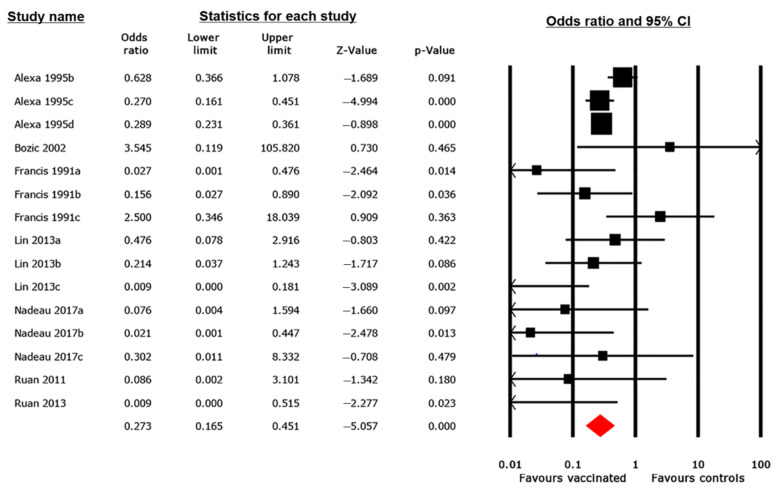
Forest plot of 15 trials assessing the effectiveness of ETEC vaccines based on mortality outcome. The pooled effect is estimated in OR with its corresponding 95% confidence interval, using a random-effects model. The black squares represent estimated ORs for each study; the size of the squares corresponds to the weight attributed to each study; horizontal solid lines represent 95% CIs; and the red diamond represents the pooled OR estimated from all studies included in the meta-analysis [4,52,53,54,57,58,61].

Seven studies, composed of 15 trials, assessed the efficacy of vaccines in preventing mortality among vaccinated pigs compared with that in controls (Figure 3). The pooled OR was 0.273 (95% CI 0.165, 0.451), indicating a statistically significant reduction in mortality risk for vaccinated pigs compared with that in controls (*p* < 0.001). However, between-study heterogeneity was significantly high (*I^2^* = 53%). To assess the reason for this heterogeneity, a subgroup analysis based on the vaccine’s target antigens was conducted. The results revealed a significant relationship (Q = 15.789, *p =* 0.007) between the observed effect size and the vaccine antigen (Table 2). Therefore, we rejected the null hypothesis and concluded that variability in target antigens among the studies contributed to the observed between-study heterogeneity. In contrast, no statistically significant difference in effect sizes between subgroups was demonstrated when the vaccine category (Q = 2.001, *p* = 0.157) and route of administration (Q = 1.806, *p* = 0.614) were used as covariates (Table S3).

#### 3.4.3. ADWG Outcome

**Figure 4:**
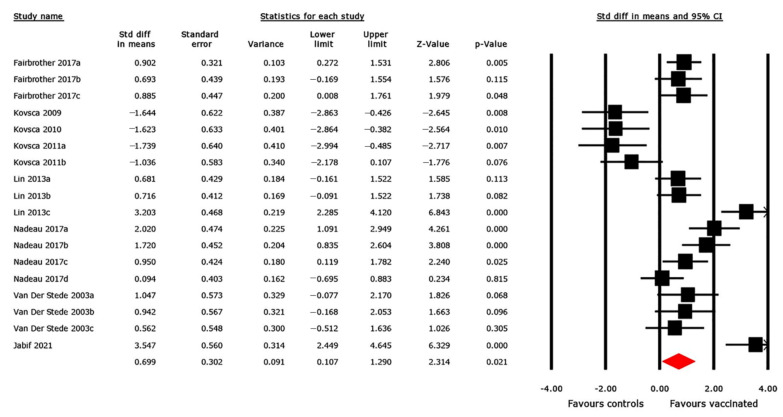
Forest plot of 18 trials assessing the effectiveness of ETEC vaccines based on daily weight gain. The pooled effect is estimated in standardized mean difference (SMD) with its corresponding 95% confidence interval, using a random-effects model. The black squares represent estimated SMDs for each study; the size of the squares corresponds to the weight attributed to each study; horizontal solid lines represent 95% CIs; and the red diamond represents the pooled SMD estimated from all studies included in the meta-analysis [4,7,9,55,56,61,63,64].

Eight studies, with a total of 18 trials, evaluated the effect of vaccination on ADWG outcome. The effect size was estimated as the SMD between vaccinated and non-vaccinated pigs (Figure 4). The pooled SMD was 0.699 (95% CI 0.107, 1.290), revealing a statistically significant increase in ADWG in vaccinated pigs compared with that in controls (*p* = 0.021). The heterogeneity between studies was considerably high (*I^2^* = 86%). The results of the subgroup analysis revealed a significant association between effect size and vaccine target antigens (Table 3). The Q statistic, which tests the null hypothesis that all subgroups share a common effect, was statistically significant (Q = 18.931, *p* = 0.001). Thus, the null hypothesis was rejected and the difference in effect size between subgroups was confirmed due to the vaccine target antigen. Using the vaccination route and vaccine category as covariates, an insignificant difference in effect size between subgroups was detected. The Q statistic was 0.794 (*p* = 0.373) for the vaccine category and 1.674 (*p* = 0.433) for the route of administration (Table S4).

#### 3.4.4. Publication Bias

**Figure 5:**
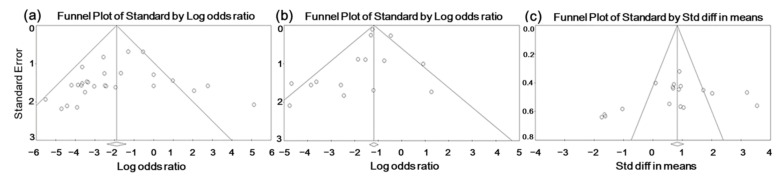
(**a**) Funnel plot for publication bias for the diarrhea outcome; (**b**) funnel plot for publication bias for the mortality outcome; and (**c**) funnel plot for publication bias for the average daily weight gain outcome. Each white circle represents an individual study, and the white triangle represents the specific region where 95% of the data points would belong to in the absence of publication bias.

Larger studies that report relatively high effects are more likely to be published, as they tend to be statistically significant, than smaller studies with low effects. To assess the presence of publication bias, funnel plots were plotted with the effect size on the *x*-axis and standard error on the *y*-axis. Visual inspection of the plots showed an asymmetric distribution of the studies across all outcomes (diarrhea, mortality, and ADWG), suggesting the existence of publication bias (Figure 5). Since funnel plot analysis is highly subjective, Egger’s regression test was applied to confirm the relationship between the effect size and sample size. The one-tailed and two-tailed analyses yielded *p*-values of 0.128 and 0.256 for diarrhea, 0.165 and 0.330 for mortality, and 0.110 and 0.219 for ADWG, respectively (Table S5). We failed to reject the null hypothesis as the *p*-values were not statistically significant; thus, the presence of publication bias was not confirmed.

### 3.5. Bayesian NMA

#### 3.5.1. Summary of the Network Geometry

**Figure 6:**
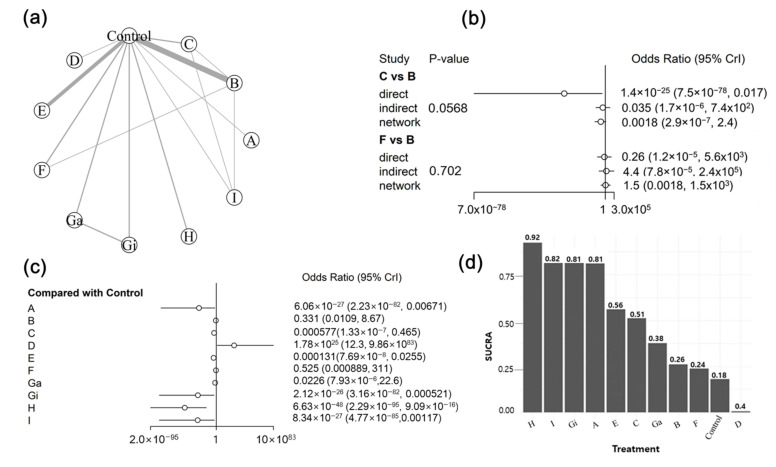
Results of the network meta-analysis of ETEC vaccines on diarrhea outcome. (**a**) Network graph for eligible treatment comparisons; each node represents one type of vaccine, the line connecting two nodes (edge) represents the evidence from direct treatment comparisons, and the edge thickness indicates how often treatments were compared in the primary trials. (**b**) Node split results for the assessment of inconsistency; (**c**) forest plot presenting the effectiveness of vaccines when compared with the control group; (**d**) surface under the cumulative ranking curve (SUCRA) for each vaccine from the best to worst.

**Figure 7:**
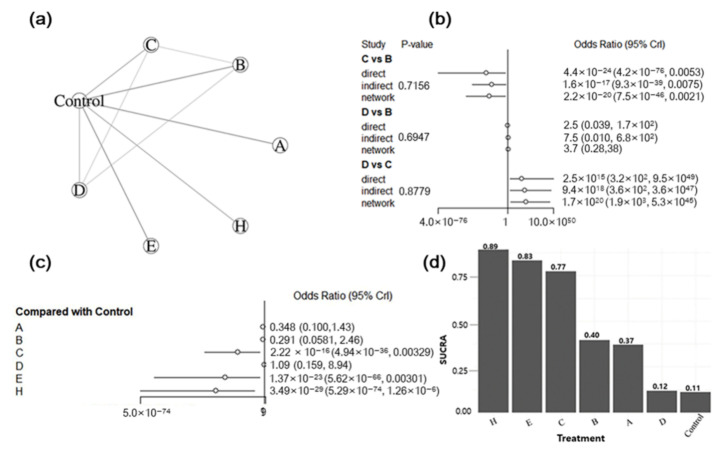
Results of the network meta-analysis of ETEC vaccines on mortality outcome. (**a**) Network graph for eligible treatment comparisons; each node represents one type of vaccine, the line connecting two nodes (edge) represents the evidence from direct treatment comparisons, and the edge thickness indicates how often treatments were compared in the primary trials. (**b**) Node split results for the assessment of inconsistency; (**c**) forest plot presenting the effectiveness of vaccines when compared with the control group; (**d**) SUCRA for each vaccine from the best to worst.

**Figure 8:**
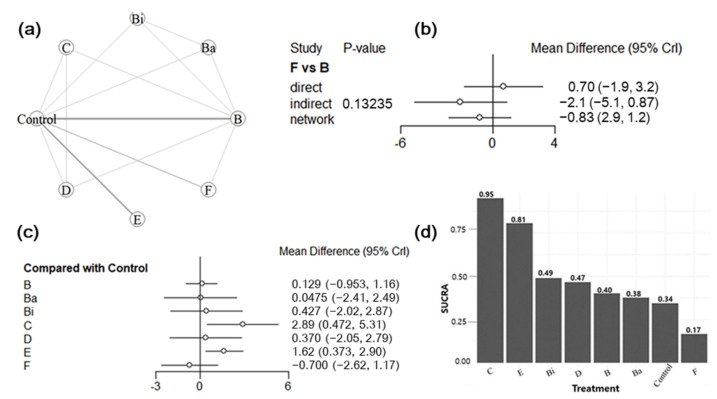
Results of the network meta-analysis of ETEC vaccines on average daily weight gain (ADWG) outcome. (**a**) Network graph for eligible treatment comparisons; each node represents one type of vaccine, the line connecting two nodes (edge) represents the evidence from direct treatment comparisons, and the edge thickness indicates how often treatments were compared in the primary trials. (**b**) Node split results for the assessment of inconsistency; (**c**) forest plot presenting the effectiveness of vaccines when compared with the control group; (**d**) SUCRA for each vaccine from the best to worst.

In the network of interventions, vaccines were grouped based on their active ingredients (i.e., vaccine target antigens). The decision to group vaccines based on their target antigens was based on the general knowledge that an effective vaccine would target both ETEC virulent determinants (i.e., fimbriae and enterotoxins) due to their crucial role in the occurrence of ETEC-associated PWD. Furthermore, because most of the studies included in the analysis assessed the efficacy of the candidate vaccines, we aimed to generate empirical evidence on the effectiveness of different target antigens in preventing PWD for future vaccine development. For diarrhea outcome, 23 trials were included in the analysis. Of these, three were three-arm trials, one was a four-arm trial, and the remainder were two-arm trials. Among the 13 studies included in the mortality outcome, one study was a four-arm trial and the remainder were two-arm trials. For the ADWG, 13 studies were analyzed: one was a three-arm trial, two were four-arm trials, and the remainder were two-arm trials. The vaccine target antigens used for each outcome are shown in Table 4. In the treatment network, the placebo and non-treated groups were combined into a single group called “control” and served as comparators. Graphical representations of the network for treatment comparisons are shown in Figure 6a, Figure 7a and Figure 8a. Eleven, seven, and eight treatments for the prevention of PWD in pigs were compared for diarrhea, mortality, and ADWG outcomes, respectively.

#### 3.5.2. Assessment of Inconsistency

On using the node-splitting method, no evidence was found of inconsistency between the direct and indirect effect estimates for all outcomes (Figure 6b, Figure 7b and Figure 8b). There was no inconsistency in the direct and indirect comparisons between antigens C and B (*p* = 0.0568), F and B (*p =* 0.702) for the diarrhea outcome, and F and B (*p* = 0.13235) for the ADWG outcome. Similarly, no evidence of inconsistency was detected between C and B (*p* = 0.7156), D and B (*p* = 0.6947), and D and C (*p* = 0.8779) for mortality outcomes. These results show no confirmation of the inconsistency. Node-splitting analysis was not performed for all treatment comparisons because of a lack of direct comparative studies.

#### 3.5.3. Synthesis of NMA Results

Forest plots summarizing the NMA results are presented in Figure 6c, Figure 7c and Figure 8c. The pooled effects were estimated using ORs for diarrhea and mortality outcomes and the MD for ADWG. All effect sizes were estimated with their corresponding 95% CrIs. Data from the Bayesian NMA revealed that all vaccine target antigens, except for that in treatment D (LT antigen), significantly reduced the incidence of diarrhea (Figure 6c) and mortality (Figure 7c) in vaccinated pigs compared with that in controls. Using the control as a reference treatment, vaccine H (F4 + F18 + LT) demonstrated the highest efficacy in preventing PWD with ORs of 6.63 × 10^−48^ (2.29 × 10^−95^, 9.09 × 10^−16^) and 3.49 × 10^−29^ (5.29 × 10^−74^, 1.26 × 10^−6^) for diarrhea and mortality outcomes, respectively. Similarly, all vaccine target antigens significantly improved ADWG (Figure 8c) among vaccinated pigs compared with that in controls, except for those in treatment F (F18ac antigen). Vaccine C (F4 + LT) resulted in the greatest improvement in ADWG, with an MD of 2.89 (0.472, 5.31). The cumulative probabilities of being the best treatment (SUCRA score) indicated that treatment H (F4 + F18 + LT) was the best regimen to prevent diarrhea (92%) and mortality (89%) outcomes, whereas treatment D (LT enterotoxin) was ranked the worst in preventing diarrhea (0.4%) and mortality (12%) outcomes. The second-best target antigen was I (F4 + LT + ST) for diarrhea outcome (82%) and antigen E (F4 + F18) for mortality outcome (83%). On the other hand, treatment C (F4 + LT) was ranked the most efficacious treatment for increasing ADWG (95%), followed by antigen E (F4 + F18) with a score of 81%, whereas vaccine F (F18ac fimbriae) was the least efficacious (17%). The treatment rankings for all outcomes are presented in Figure 6d, Figure 7d and Figure 8d. The overall findings indicated that the vaccinated pigs benefitted substantially from the vaccines, whereas unvaccinated control pigs suffered from diarrhea, mortality, and weight loss. Furthermore, vaccines targeting both fimbriae and enterotoxins demonstrated better efficacy than vaccines only targeting either fimbriae or enterotoxins.

## 4. Discussion

Swine vaccination is perceived as the most effective preventive approach to counteract PWD in the pig industry [23,64,65]. Owing to the levels of mortality and morbidity associated with ETEC-inflicted diarrhea, as well as the scope of the global disease burden, several initiatives to develop effective vaccines have been launched. Several experiment trials have shown that the microbes actively colonizing the mucosal surfaces of the intestines can effectively stimulate the secretory immune system of the gut to produce appropriate antibodies [66,67]. Following immunization, the vaccine induces mucosal immunity and the production of antigen-specific serum IgA antibodies, which inhibit ETEC adherence to specific receptors on enterocytes [3,68]; thus, blocking the bacteria colonization of the small intestines and protecting against ETEC-associated PWD [15,69]. In this study, we evaluated the effectiveness of commercially available and candidate vaccines against ETEC-associated PWD in pigs. The effectiveness of the vaccines was assessed based on three clinical outcomes: mortality, diarrhea, and ADWG. Subsequently, a simultaneous comparison of vaccines was conducted using Bayesian NMA to generate evidence-based data on the most effective vaccine based on their target antigens.

The MA results were generated using a random-effects model, which is an analytical method that assumes that the true effect varies across studies [31]. This assumption was deemed necessary because of the considerable variability in the implementation of interventions among the studies included in this systematic review. The overall pooled results of the pairwise MA showed that immunization significantly reduced the odds of diarrhea and mortality and increased ADWG among vaccinated pigs compared with that in controls (Figure 2, Figure 3 and Figure 4), although the between-study heterogeneity was significantly high for all outcomes (*I*^2^ > 50%). It is broadly perceived that effective protection against PWD could be achieved only with vaccines that induce both protective anti-adhesion and antitoxin immunity due to immunologically varied fimbriae and enterotoxins expressed by the ETEC strains that cause PWD [23]. Therefore, to assess whether there was any association between the effect size and vaccine target antigens, a subgroup analysis was conducted. The subgroup analysis findings confirmed a significant relationship between the effect size and vaccine target antigens for all outcomes (Table 1, Table 2 and Table 3). Thus, we concluded that the observed difference among vaccine effects was due to variability in the antigens targeted by the vaccines.

Regarding NMA, the findings revealed that all target antigens were effective in preventing PWD in pigs, except for antigen D (LT enterotoxin). The SUCRA ranking indicated that antigen H (combination of F4ac, F18ac fimbriae, and LT enterotoxin) was the best regimen to reduce the incidence of diarrhea and mortality, whereas antigen D (LT enterotoxin) was ranked the worst. The observed differences in the efficacy of ETEC antigens in stimulating immune response can be multifactorial. The adherence of ETEC to the intestinal enterocytes in pigs is the initial and most critical step in the pathogenesis of ETEC [70]; hence, vaccines that induce neutralizing antibodies to block F4 and F18 adhesion to the intestinal mucosa and halt enterotoxin secretion would effectively protect piglets from PWD [65,71]. Similar results have also been reported in different studies where oral immunization with a live attenuated *E. coli* strain expressing a holotoxin-structured adhesin-toxoid fusion (1FaeG-FedF-LTA_2_: 5LTB) or with a tripartite fusion (FaeG-FedF-LT192A2: B) of ETEC elicited antibodies that neutralized toxins, inhibited adherence of F4 and F18 fimbriae, and protected pigs against ETEC infection [57,58].

One potential reason for low levels of protection after LT vaccination is that the toxin is not required for bacterial colonization, although it may enhance colonization in some cases [72]. Therefore, colonization of the intestinal epithelium is likely to continue in the presence of anti-LT antibodies. At some point during colonization, the amount of ETEC-produced LT may exceed the amount of antibodies produced by the host’s immune cells to neutralize the toxin, resulting in diarrhea [61]. Thus, complete immune-mediated protection against ETEC needs the production of antibodies that inhibit pathogen colonization in addition to enterotoxin neutralization [52,66]. In previously reported cases, diarrhea could occur even in the absence of enterotoxins after ETEC attachment to and colonization of the intestinal epithelial cells [73,74]. Consequently, even high anti-LT antibody titers may not prevent diarrhea if bacterial colonization has already occurred [52,61]. For the ADWG outcome, antigen C (combination of F4ac fimbriae with LT enterotoxin) was the best regimen to improve daily weight gain, whereas antigen F (F18ac fimbriae) was ranked the worst. This difference in immunogenicity is due to the role of F4 and F18 fimbriae, and LT enterotoxin in inducing the immune response. The F4 fimbriae have a major structural subunit FaeG, which is highly immunogenic and is present in multiple copies on a single fimbriae [19,75], whereas the F18 ETEC presents a minor structural subunit FedF that serves as an adhesive subunit and is not effective in inducing anti-F18 antibody response [19,23,76,77]. The LT enterotoxin possesses great adjuvanticity that enhances vaccine-specific systemic and mucosal immune responses following mucosal or parenteral delivery [76,78]. Hence, a bivalent vaccine combining both F4 fimbriae and LT enterotoxin would result in better protection than that provided by using only F18 fimbriae as it would produce both anti-adhesin and anti-enterotoxin antibodies. Similar findings have been reported in several other studies where immunization of piglets with a vaccine encoding for F4 fimbriae and enterotoxin antigens conferred complete protection against PWD and improved the daily weight gain in a virulent ETEC challenge model [60,61], whereas vaccination with F18 fimbriae alone did not prove as effective [76,79].

Regarding vaccine delivery, different studies have suggested that the oral route is the most logical route to deliver ETEC vaccines because vaccines administered through this route can rapidly induce the mucosal antibody response with secretory IgA secretion, which is critical for preventing bacterial colonization and neutralizing enterotoxins [19,76]. This immune response is hardly achieved by parenteral vaccines as they tend to induce systemic immunity rather than mucosal immunity [19,23]. In this study, the results of subgroup analysis did not confirm a significant relationship between the observed effect size and route of vaccine delivery (i.e., oral, intranasal, parenteral, or combined routes). However, because of an insufficient number of studies within the subgroups, the low power of statistical tests in detecting the relationship between this covariate and the observed effect size cannot be ruled out. Similarly, no statistically significant difference was observed in the effect sizes between studies that used commercial vaccines and those that used experimental candidate vaccines. Although funnel plots showed an asymmetric distribution of studies within the graphs, the risk of publication bias was not confirmed. The results of Egger’s regression test yielded non-significant *p*-values for all outcomes (*p* > 0.05); thus, we failed to reject the null hypothesis that there is no risk of publication bias. For future studies, experimental trials may need to be conducted in field settings under natural exposure to ETEC infections to determine the magnitude of efficacy of vaccines with the various combinations of the target antigens and evaluate its side effects to guide clinical decision-making by stakeholders and veterinary clinicians.

There were a few limitations to this systematic review and NMA. Regarding transitivity, there was reasonable variability in the duration of treatment protocol among the trials. This variability might have caused differences in treatment efficacy; thus, lowering the statistical power. Although the findings of both pairwise and NMA support the use of vaccines for preventing PWD in swine, the adverse effects of these vaccines are still uncertain. The presence of inconsistency was not confirmed; however, node-splitting analysis could not be performed for all treatment comparisons because of the lack of direct evidence from randomized controlled trials. Another limitation is the unexpected scarcity of studies conducted under natural field settings with an adequately blinded assessment of clinically important outcomes.

## 5. Conclusions

In conclusion, the findings of this study support immunization of pigs to prevent PWD. However, the type of target antigen determines the magnitude of the vaccine efficacy, as discussed in this systematic review and MA. Since ETEC fimbriae and enterotoxins are genetically and immunologically heterogeneous, immunity induced by one fimbriae or enterotoxin cannot protect against heterologous ETEC strains. Therefore, a multivalent vaccine that targets both fimbriae and enterotoxins should be prioritized to combat post-weaning diarrhea in pigs.

## Figures and Tables

**Table 1:** Subgroup analysis of diarrhea outcome based on vaccine antigens.

Groups	Number of Studies	Effect Size and 95% Confidence Interval			Test of Null (Two-Tail)		Heterogeneity		
		Point Estimate	Lower Limit	Upper Limit	Z-Value	*p*-Value	Q-Value	df (Q)	*p*-Value
F18 + LT + ST	1	0.030	0.000	1.896	−1.658	0.097	19.162	8	0.014
F18	2	1.000	0.076	13.160	0.000	1.000			
F4	9	0.388	0.118	1.270	−1.565	0.117			
F4 + F18	6	0.041	0.009	0.194	−4.040	0.000			
F4 + F18 + LT	2	0.013	0.000	0.414	−2.465	0.014			
F4 + F5 + F6 + F41	4	0.098	0.018	0.528	−2.701	0.007			
F4 + LT	2	0.014	0.001	0.183	−3.248	0.001			
F4 + LT + ST	1	0.004	0.000	0.346	−2.427	0.015			
LT	1	7.909	0.133	469.971	0.992	0.321			
Overall	28	0.128	0.063	0.257	−5.758	0.000			

F18 = F18ac fimbriae. F4 = F4ac fimbriae. F5 = F5 fimbriae. df = degree of freedom. F6 = F6 fimbriae. F41 = F41 fimbriae. LT = heat labile enterotoxin. ST = heat stable enterotoxin.

**Table 2:** Subgroup analysis of mortality outcome based on types of vaccine antigens.

Groups	Number of Studies	Effect Size and 95% Confidence Interval			Test of Null (Two-Tail)		Heterogeneity		
		Point Estimate	Lower Limit	Upper Limit	Z-Value	*p*-Value	Q-Value	df (Q)	*p*-Value
F18 + LT + ST	3	0.351	0.229	0.536	−4.835	0.000			
F4	3	0.265	0.078	0.901	−2.127	0.033			
F4 + F18	3	0.073	0.012	0.460	−2.786	0.005			
F4 + F18 + LT	2	0.031	0.002	0.483	−2.481	0.013			
F4 + LT	2	0.016	0.002	0.132	−3.840	0.000			
LT	2	1.022	0.252	4.155	0.031	0.975			
Overall	15	0.301	0.208	0.435	−6.385	0.000	15.789	5	0.007

**Table 3:** Subgroup analysis of the daily weight gain outcome based on vaccine antigen types.

Groups	Number of Studies	Effect Size and 95% CI					Test of Null (Two-Tail)		Heterogeneity		
		Point Estimate	Standard Error	Variance	Lower Limit	Upper Limit	Z-Value	*p*-Value	Q-Value	df(Q)	*p*-Value
F18	2	−1.334	0.754	0.568	−2.811	0.144	−1.769	0.077			
F4	9	0.332	0.339	0.115	−0.332	0.996	0.980	0.327			
F4 + F18	5	1.608	0.445	0.198	0.737	2.480	3.618	0.000			
F4 + LT	1	3.203	0.997	0.993	1.250	5.156	3.214	0.001			
LT	1	0.681	0.979	0.958	−1.238	2.599	0.695	0.487			
Overall	18	0.718	0.239	0.057	0.250	1.185	3.010	0.003	18.931	4	0.001

**Table 4:** List of target antigens included in the vaccines compared in the Bayesian NMA.

Outcome	Vaccine Antigen	Antigen ID
Diarrhea	F18 + LT + ST	A
	F4	B
	F4 + LT	C
	LT	D
	F4 + F18	E
	F18ac	F
	F4ab + F4ac + F5 + F6 + F41 F4ab + F4ac + F4ad + F5 + F6 + F41	Gi Ga
	F4 + F18 + LT	H
	F4 + LT + ST	I
Mortality	F18 + LT + ST	A
	F4	B
	F4 + LT	C
	LT	D
	F4 + F18	E
	F4 + F18 + LT	H
Average daily weight gain (ADWG)	F4	B
	F4 + LT	C
	LT	D
	F4 + F18	E
	F18ac	F
	F4 + D3	Bi
	F4 + Cpg	Ba

## Data Availability

All data used or analyzed during this study are included in this article and its Supplementary Information Files.

## Supplementary Materials

The following supporting information can be downloaded at: https://www.mdpi.com/article/10.3390/ani12162136/s1, Table S1: Characteristics of 17 studies included in the systematic review and Bayesian network meta-analysis (NMA). Each row in the table corresponds to one trial. Different trials from the same publication are indicated by the letters a, b, c, and d; Table S2: Subgroup analysis of diarrhea outcomes by vaccine category (A), and vaccine route of administration (B). Studies were classified into two (commercial and experimental) and three (oral, parenteral, and combined) groups for the vaccine category and vaccination route, respectively. Results were generated using a mixed-effects model; Table S3: Subgroup analysis of mortality outcome by vaccine category (A), and route of administration (B). Studies were classified into two (commercial and experimental) and four (oral, parenteral, intranasal, and combined) groups for the vaccine category and route of administration, respectively. Results were generated using a mixed-effects model; Table S4: Subgroup analysis of the average daily weight gain outcome by vaccine category (A), and route of administration (B). Studies were classified into two (commercial and experimental) and three (oral, parenteral, and intranasal) groups for the vaccine category and vaccination route, respectively. Results were generated using a mixed-effects model; Table S5: Egger’s regression test results for publication bias. Summary description table of quantitative estimates of publication bias for diarrhea, mortality, and daily weight gain outcomes. An insignificant relationship was observed between the effect size and sample size for all outcomes; Table S6: PRISMA 2020 checklist; Table S7: PRISMA NMA checklist of items to include when reporting a systematic review involving an NMA.
