# Supplementary material for: Evidence-Based Approaches for Determining Effective Target Antigens to Develop Vaccines against Post-Weaning Diarrhea Caused by Enterotoxigenic Escherichia coli in Pigs: A Systematic Review and Network Meta-Analysis
Source: Animals (Basel). 2022 Aug 19;12(16):2136. doi: 10.3390/ani12162136 (PMC9405027; doi:10.3390/ani12162136)
Supplement: Supplementary file 1 [file animals-12-02136-s001.zip › Table S5.pdf]

Egger's regression test results for publication bias

Items	Outcomes		
	**Diarrhea**	**Mortality**	**DWG**
Intercept	-0.98208	-0.48462	-4.20753
Standard error	0.84484	0.47899	3.29015
95% Lower limit	-2.71868	-1.51942	-11.18234
95% Upper limit	0.75452	0.55017	2.76727
t-value	1.16244	1.01177	1.27883
df	26	13	16
P-value (1-tailed)	0.12781	0.16506	0.1096
P-value (2-tailed)	0.25562	0.33011	0.2192
