# Supplementary material for: Evidence-Based Approaches for Determining Effective Target Antigens to Develop Vaccines against Post-Weaning Diarrhea Caused by Enterotoxigenic Escherichia coli in Pigs: A Systematic Review and Network Meta-Analysis
Source: Animals (Basel). 2022 Aug 19;12(16):2136. doi: 10.3390/ani12162136 (PMC9405027; doi:10.3390/ani12162136)
Supplement: Supplementary file 1 [file animals-12-02136-s001.zip › Table S1.pdf]

Characteristics of 17 studies included in a systematic review and Bayesian network meta-analysis.

Author	Trial	Study design	Vaccine category	Vaccine dose	Weaning age	Time of vaccination	Vaccination route	Adjuvant	Control	Time of challenge	Sample size	Follow-up	Extracted outcomes
**Alexa 1995**	Alexa 1995a	Experimental study	Experimental (Vaccine Candidate)	**Inact:** 1ml/pig (2x10^9CFU/ml)<br>**Live:** 7ml/pig (2x10^9CFU/ml)	35 days	34 & 35 daysold	**Inact:** Parenteral (IM);<br>**Live:** Oral	No adjuvant	No vaccine	**7dpsi** {ETEC strain No. 8943 (2x10^9 CFU/ml); 7ml/pig}	**Vacc:** 6 pigs;<br>**Ctrl:** 4 pigs	7dpc	Diarrhea
	Alexa 1995b	Field study<br>**(Farm A)**	Experimental (Vaccine Candidate)	**Inact:** 1ml/pig (2x10^9CFU/ml)<br>**Live:** 7ml/pig (2x10^9CFU/ml)	35 days	34 & 35 daysold	**Inact:** Parenteral (IM);<br>**Live:** Oral	No adjuvant	No vaccine	**NA**	**Vacc:** 222 pigs;<br>**Ctrl:** 232 pigs	14dpw	Mortality
	Alexa 1995c	Field study<br>**(Farm B)**	Experimental (Vaccine Candidate)	**Inact:** 1ml/pig (2x10^9CFU/ml)<br>**Live:** 7ml/pig (2x10^9CFU/ml)	35 days	34 & 35 daysold	**Inact:** Parenteral (IM);<br>**Live:** Oral	No adjuvant	No vaccine	**NA**	**Vacc:** 255 pigs;<br>**Ctrl** : 274 pigs	14dpw	Mortality
	Alexa 1995d	Field study<br>**(Farm C)**	Experimental (Vaccine Candidate)	**Inact:** 1ml/pig (2x10^9CFU/ml)<br>**Live:** 7ml/pig (2x10^9CFU/ml)	35 days	34 & 35 daysold	**Inact:** Parenteral (IM);<br>**Live:** Oral	No adjuvant	No vaccine	**NA**	**Vacc:** 3692 pigs;<br>**Ctrl:** 6301 pigs	14dpw	Mortality
**Bozic et al., 2002**	Bozic et al. 2002	Experimental study	Experimental (Vaccine Candidate)	60ml/pig (10^10CFU)	4 weeks	2dpw	Oral	No adjuvant	TSB (Trypticase soy broth)	**7dpi** {F4ac+ETEC strain (0149:K91:F4 ac: 987P:Hly+++ LT +STb+)}	**Vacc:** 6 pigs;<br>**Ctrl:** 6 pigs	7dpc	Diarrhea;<br>Mortality

**Fairbrother et al., 2017**	Fairbrother et al. 2017a	Experimental study	Commercial	150ml/pig (5.9x107 CFU)	18-19 days	1dpw	Oral	No adjuvant	150 ml filtered water	**3dpi** {F4-ETEC EcL8559 O149 challenge strain}	**Vacc:** 20 pigs; **Ctrl:** 20 pigs	5dpc	Diarrhea; ADWG
	Fairbrother et al. 2017b	Experimental study	Commercial	150ml/pig (1.3x108 CFU)	18-19 days	1dpw	Oral	No adjuvant	150 ml filtered water	**7dpi** {F4-ETEC EcL8559 O149 challenge strain}	**Vacc:** 10 pigs; **Ctrl:** 10 pigs	3dpc	Diarrhea; ADWG
	Fairbrotheret al. 2017c	Experimental study	Commercial	150ml/pig (1.3x108 CFU)	18-19 days	1dpw	Oral	No adjuvant	150 ml filtered water	**21dpi** {F4-ETEC EcL8559 O149 challenge strain}	**Vacc:** 10 pigs; **Ctrl:** 10 pigs	3dpc	Diarrhea; ADWG
**Francis et al., 1991**	Francis et al. 1991a	Experimental study	Experimental (Vaccine Candidate)	3ml/pig (10^9 CFU)	10 days	14 & 19 days old	Oral	No adjuvant	ETEC strain G58-1 (0101:K28:NM)	**2wpi** {ETEC strain 3030-2 (0157:K88, LT+, STb+)}	**Vacc:** 33 pigs; **Ctrl:** 31 pigs	2dpc	Diarrhea; Mortality
	Francis et al. 1991b	Experimental study	Experimental (Vaccine Candidate)	3ml/pig (10^9 CFU)	10 days	14 & 19 days old	Oral	No adjuvant	ETEC strain G58-1 (0101:K28:NM)	**2wpi** {ETEC strain 3030-2 (0157:K88, LT+, STb+)}	**Vacc:** 18 pigs; **Ctrl:** 18 pigs	2dpc	Diarrhea; Mortality
	Francis et al. 1991c	Experimental study	Experimental (Vaccine Candidate)	3ml/pig (10^9 CFU)	10 days	14 & 19 days old	Oral	No adjuvant	ETEC strain G58-1 (0101:K28:NM)	**2wpi** {ETEC strain 3030-2 (0157:K88, LT+, STb+)}	**Vacc:** 14 pigs; **Ctrl:** 6 pigs	2dpc	Diarrhea; Mortality
**J.Lee et al., 2012**	J.Lee et al. 2012	Experimental study	Experimental (Vaccine Candidate)	10ml/pig (2x10^10CFU) (vaccinated pregnant sows)	28 days	Vaccinated pregnant sows (8 & 11 weeks of pregnancy)	Oral	No adjuvant	10ml PBS	**5 weeks of age** {Hemolytic wild type E.coli isolates JOL500 and JOL599 containing F18 and F4 genes respectively}	**Vacc:** 21 pigs; **Ctrl:** 23 pigs	21dpc	Diarrhea
**Jabif et al., 2021**	Jabif et al. 2021	Experimental study	Experimental (Vaccine Candidate)	2ml/pig	28 days	33 & 43 days of age	Oral	No adjuvant	2ml/pig saline solution	**46 & 47 days of age** {Two wild-type Enterotoxigenic E. coli (ETEC) field isolates, VBTEColi-1 and VBTEColi-2. (108 cfu/mL). 2ml/pig}	**Vacc:** 15 pigs; **Ctrl:** 15 pigs	10dpc	Diarrhea; ADWG

**Kovsca et al., 2009**	Kovsca et al. 2009	Experimental study	Experimental (Vaccine Candidate)	60ml/pig (10^10CFU F18ac)	4 weeks	2dpw	Oral	Levamisole	Saline solution	**7dpi** {F4ac+ ETEC strain 11-800/1/94 (0149:K91:F4ac: 987P: Hly+LT+STb+)}	**Vacc:** 5 pigs; **Ctrl:** 5 pigs	7dpc	Diarrhea; ADWG
**Kovsca et al., 2010**	Kovsca et al. 2010	Experimental study	Experimental (Vaccine Candidate)	60ml/pig (10^10CFU F4ac)	4 weeks	2dpw	Oral	Levamisole	5ml saline	**7dpi** {F4ac+ strain ETEC 11-800/1/94 (0149:K91:Fac:987P: Hly+LT+STb+)}	**Vacc:** 5 pigs; **Ctrl:** 5 pigs	7dpc	Diarrhea; ADWG
**Kovsca et al., 2011**	Kovsca et al. 2011a	Experimental study	Experimental (Vaccine Candidate)	60ml/pig (10^10 CFU F4ac+)	4 weeks	2dpw	Oral	Levamisole	5ml saline	**7dpi** {10^10CFU/ml: F4ac+ETEC strain 11-800/1/94 (019:K91:F4ac:987P: Hly+LT+STb+)}	**Vacc:** 5 pigs; **Ctrl:** 5 pigs	7dpc	Diarrhea; ADWG
	Kovsca et al. 2011b	Experimental study	Experimental (Vaccine Candidate)	60ml/pig (10^10CFU F18ac+)	4 weeks	2dpw	Oral	Levamisole	5ml saline	**7dpi** {10^10CFU/ml: F4ac+ETEC strain 11-800/1/94 (019:K91:F4ac:987P: Hly+LT+STb+)}	**Vacc:** 5 pigs; **Ctrl:** 5 pigs	7dpc	Diarrhea; ADWG
**Lee et al.,2012**	Lee et al. 2012a	Experimental study	Experimental (Vaccine Candidate)	10ml/pig (2x10^10 CFU): vaccinated pregnant sows	21 days	Vaccinated pregnant sows (8 & 11 weeks of pregnancy)	Oral	No adjuvant	10ml PBS	**1 week of age** {**1.** JOL489 (Wild type F5+, F41+, LT+, stx2+ ETEC); **2.** JOL564 (Wild type F6+, stx1+, stx2+ ETEC); **3.** JOL599 (Wild type F4+, LT+, STa+, STb+, EAST 1+ ETEC). 3ml (1x10^9CFU/ml) of each challenge strain}	**Vacc:** 18 pigs; **Ctrl:** 18 pigs	21dpc	Diarrhea
	Lee et al. 2012b	Experimental study	Commercial	NR	21 days	Vaccinated pregnant sows (11 & 14 weeks of pregnancy)	Parenteral (IM)	No adjuvant	10ml PBS	**1 week of age** {**1.** JOL489 (Wild type F5+, F41+, LT+, stx2+ ETEC); **2.** JOL564 (Wild type F6+, stx1+, stx2+ ETEC); **3.** JOL599 (Wild type F4+, LT+, STa+, STb+, EAST 1+ ETEC).	**Vacc:** 17 pigs; **Ctrl:** 18 pigs	21dpc	Diarrhea

										3ml (1x10^9CFU/ml) of each challenge strain}			
	Lee et al. 2012c	Experimental study	Experimental (Vaccine Candidate)	10ml/pig (2x10^10 CFU): vaccinated pregnant sows	21 days	Vaccinated pregnant sows (8 & 11 weeks of pregnancy)	Oral	No adjuvant	10ml PBS	**3 weeks of age** {**1.** JOL489 (Wild type F5+, F41+, LT+, stx2+ ETEC); **2.** JOL564 (Wild type F6+, stx1+, stx2+ ETEC); **3.** JOL599 (Wild type F4+, LT+, STa+, STb+, EAST 1+ ETEC). 3ml (1x10^9CFU/ml) of each challenge strain}	**Vacc:** 19 pigs; **Ctrl:** 16 pigs	21dpc	Diarrhea
	Lee et al. 2012d	Experimental study	Commercial	NR	21 days	Vaccinated pregnant sows (11 & 14 weeks of pregnancy)	Parenteral (IM)	No adjuvant	10ml PBS	**3 weeks of age** {**1.** JOL489 (Wild type F5+, F41+, LT+, stx2+ ETEC); **2.** JOL564 (Wild type F6+, stx1+, stx2+ ETEC); **3.** JOL599 (Wild type F4+, LT+, STa+, STb+, EAST 1+ ETEC). 3ml (1x10^9CFU/ml) of each challenge strain}	**Vacc:** 19 pigs; **Ctrl:** 16 pigs	21dpc	Diarrhea
**Lin et al., 2013**	Lin et al. 2013a	Experimental study	Experimental (Vaccine Candidate)	100µg LT	NA (Gnotobiotic piglets)	10 and 17 days of age	Parenteral (IN)	No adjuvant	1ml PBS	**7dpsi** {E. coli strain 3030-2 (O157:K88ac+/LT+/STb+). 3ml TSB (2x10^9CFU)}	**Vacc:** 7 pigs; **Ctrl:** 19 pigs	4dpc	Mortality; ADWG
	Lin et al. 2013b	Experimental study	Experimental (Vaccine Candidate)	300µg K88	NA (Gnotobiotic piglets)	10 and 17 days of age	Parenteral (IN)	No adjuvant	1ml PBS	**7dpsi** {E. coli strain 3030-2 (O157:K88ac+/LT+/STb+). 3ml TSB (2x10^9CFU)}	**Vacc:** 8 pigs; **Ctrl:** 19 pigs	4dpc	Mortality; ADWG

	Lin et al. 2013c	Experimental study	Experimental (Vaccine Candidate)	300µg K88 + 100µg LT	NA (Gnotobiotic piglets)	10 and 17 days of age	Parenteral (IN)	No adjuvant	1ml PBS	**7dpsi** {E. coli strain 3030-2 (O157:K88ac+/LT+/STb+). 3mlTSB (2x10^9CFU)}	**Vacc:** 20 pigs; **Ctrl:** 19 pigs	4dpc	Mortality; ADWG
**Nadeau et al., 2017**	Nadeau et al. 2017a	Experimental study	Commercial	120ml/pig (2.8 × 108 CFU F18 and 1.6 × 108 CFU F4)	16-18 days of age	17-19 days	Oral	No adjuvant	No vaccine	**7dpi** {0138 F18-ETEC 9910297-2STM (Positive for STb, LT, East-1, Stx2e, F18ab)}	**Vacc:** 12 pigs; **Ctrl:** 12 pigs	7dpc	Diarrhea; Mortality; ADWG
	Nadeau et al. 2017b	Experimental study	Commercial	120ml/pig (2.8 × 108 CFU F18 and 1.6 × 108 CFU F4)	16-18 days of age	17-19 days	Oral	No adjuvant	No vaccine	**21dpi** {0138 F18-ETEC 9910297-2STM (Positive for STb, LT, East-1, Stx2e, F18ab)}	**Vacc:** 12 pigs; **Ctrl:** 12 pigs	7dpc	Diarrhea; Mortality; ADWG
	Nadeau et al. 2017c	Experimental study	Commercial	150ml/pig (1.1 × 108 CFU F18 and 5.8 × 107 CFU F4)	16-18 days of age	17-19 days	Oral	No adjuvant	No vaccine	**7dpi** {nalidixic acid resistant 0149F4-ETEC EcL8559 (Positive for STa, STb, LT, East-1, F4ac)}	**Vacc:** 10 pigs; **Ctrl:** 10 pigs	7dpc	Diarrhea; Mortality; ADWG
	Nadeau et al. 2017d	Experimental study	Commercial	150ml/pig (1.1 × 108 CFU F18 and 5.8 × 107 CFU F4)	16-18 days of age	17-19 days	Oral	No adjuvant	No vaccine	**21dpi** {nalidixic acid resistant 0149F4-ETEC EcL8559 (Positive for STa, STb, LT, East-1, F4ac)}	**Vacc:** 10 pigs; **Ctrl:** 9 pigs	7dpc	Diarrhea; ADWG
**Ruan et al., 2011**	Ruan et al. 2011	Experimental study	Experimental (Vaccine Candidate)	100 µl/pig (100 µg FaeG-FedF-LT 192A2:B fusion)	NA (Gnotobiotic piglets)	5 days and 2 weeks of age	Parenteral (IM)	Incomplete Freund adjuvant (Sigma)	No vaccine	**2wpsi** {3 × 109 CFU of the ETEC strain 3030-2 (K88ac/LT/STb)}	**Vacc:** 3 pigs; **Ctrl:** 3 pigs	2dpc	Diarrhea; Mortality
**Ruan et al., 2013**	Ruan et al. 2013	Experimental study	Experimental (Vaccine Candidate)	7.5x10^9 CFU (1FaeG-FedF-LT 192A2:5LTB fusion)	NA (Gnotobiotic piglets)	4-5 day of age	Oral	No adjuvant	Host strain 8795	**16 days of age** {8x10^10 CFU/ml ETEC strain 3030-2 (K88ac+/LT+/STb+)}	**Vacc:** 6 pigs; **Ctrl:** 4 pigs	1dpc	Diarrhea; Mortality
**Santiago et al., 2012**	Santiago et al. 2012a	Experimental study	Experimental (Vaccine Candidate)	3ml/pig (3x10^9 CFU F4ac+ ETEC strain 8017). (K88ac+; enterotoxin negative control; Hy-) mixed with milk replacer	NA (Gnotobiotic piglets)	5 days of age	Oral	No adjuvant	3ml Lactose broth mixed with milk replacer	**1dpi** {ETEC Strain 3030-2 (K88+/LT/STb). 3ml/ piglet (3x10^10CFU) mixed with milk replacer}	**Vacc:** 8 pigs; **Ctrl:** 9 pigs	1dpc	Diarrhea

	Santiago et al. 2012b	Experimental study	Experimental (Vaccine Candidate)	3ml/pig (3x10^9 CFU F4ac+ ETEC strain 8221). (K88ac+; LT mutant [LT(R192G)]; Hy-) mixed with milk replacer	NA (Gnotobiotic piglets)	5 days of age	Oral	No adjuvant	3ml Lactose broth mixed with milk replacer	**1dpi** {ETEC Strain 3030-2 (K88+/LT/STb). 3ml/ piglet (3x10^10CFU) mixed with milk replacer}	**Vacc:** 7 pigs; **Ctrl:** 9 pigs	1dpc	Diarrhea
	Santiago et al. 2012c	Experimental study	Experimental (Vaccine Candidate)	3ml/pig (3x10^9 CFU F4ac+ ETEC strain 8488). (K88ac+; LT mutant fused with STb [LT (R192G)-STb]; Hy-) mixed with milk replacer	NA (Gnotobiotic piglets)	5 days of age	Oral	No adjuvant	3ml Lactose broth mixed with milk replacer	**1dpi** {ETEC Strain 3030-2 (K88+/LT/STb). 3ml/ piglet (3x10^10CFU) mixed with milk replacer}	**Vacc:** 7 pigs; **Ctrl:** 9 pigs	1dpc	Diarrhea
**Srivastava etal. 2016**	Srivastava et al. 2016a	Experimental study	Experimental (Vaccine Candidate)	2mg F4 fimbriae loaded in porous tablets	4 weeks	1wpw	Oral	No adjuvant	PBS	**35 days of age** {ETEC strain GIS 26 (0149:K91:F4ac, LT+,STa+,STb+) 2x10^9CFU/ml}	**Vacc:** 4 pigs; **Ctrl:** 4 pigs	7dpc	Diarrhea;
**Van Der Stede et al.,2003**	Van der Stede et al. 2003a	Experimental study	Experimental (Vaccine Candidate)	100μg F4 fimbriae	29 days	8 & 25 days of life	Parenteral (IM)	Incomplete Freund adjuvant	PBS + IFA	**8dpsi** {F4+-ETEC strain GIS26}	**Vacc:** 5 pigs; **Ctrl:** 5 pigs	7dpc	ADWG
	Van der Stede et al. 2003b	Experimental study	Experimental (Vaccine Candidate)	100μg F4 fimbriae + 2μg of 1α,25 (OH)2D3	29 days	8 & 25 days of life	Parenteral (IM)	Incomplete Freund adjuvant	PBS + IFA	**8dpsi** {F4+-ETEC strain GIS26}	**Vacc:** 5 pigs; **Ctrl:** 5 pigs	7dpc	ADWG
	Van der Stede et al. 2003c	Experimental study	Experimental (Vaccine Candidate)	100μg of F4 fimbriae + 500μg of CpG-ODN	29 days	8 & 25 days of life	Parenteral (IM)	Incomplete Freund adjuvant	PBS + IFA	**8dpsi** {F4+-ETEC strain GIS26}	**Vacc:** 5 pigs; **Ctrl:** 5 pigs	7dpc	ADWG

Each row in this table corresponds to 1 trial. Different trials from the same publication are indicated by letters a, b, c and d

Abbreviations: Inact: Inactivated vaccine; **Live**: Live attenuated vaccine; **IM**: Intramuscular; **dpsi:** day post-secondary immunization; **NA:** Not applicable; **Vacc**: Vaccinated; **Ctrl**: Control; **dpc:** day post challenge; **dpw:** day post weaning; **CFU**: Colony Forming Unit; **dbw:** day before weaning; **NR**: Not reported; **dpi:** day post immunization; **dppi**: day post primary immunization; **TSB:** Trypticase soy broth; **ADWG**: average daily weight gain; **wpi:** week post immunization; **PBS:** phosphate buffered saline; **IN:** Intranasal; **dpsi:** day post-secondary immunization; **LT:** Heat labile enterotoxin; **wpw:** week post weaning; **wpti**: week post third immunisation; **CT:** Cholera toxin; **IFA**: Incomplete Freund adjuvant
