# Supplementary material for: Evidence-Based Approaches for Determining Effective Target Antigens to Develop Vaccines against Post-Weaning Diarrhea Caused by Enterotoxigenic Escherichia coli in Pigs: A Systematic Review and Network Meta-Analysis
Source: Animals (Basel). 2022 Aug 19;12(16):2136. doi: 10.3390/ani12162136 (PMC9405027; doi:10.3390/ani12162136)
Supplement: Supplementary file 1 [file animals-12-02136-s001.zip › Table S2.pdf]

A. Subgroup analysis of diarrhea outcome by vaccine category

Groups	Number of studies	Effect size and 95% C.I			Test of null (2-Tail)		Heterogeneity		
		Point estimate	Lower limit	Upper limit	Z-value	P-value	Q-value	df (Q)	P-value
**Commercial**	9	0.102	0.026	0.398	-3.289	0.001			
**Experimental**	19	0.137	0.050	0.381	-3.817	0.000			
**Overall**	**28**	**0.124**	**0.055**	**0.279**	**-5.027**	**0.000**	**0.115**	**1**	**0.734**

B. Subgroup analysis of diarrhea outcome based on the vaccination route

Groups	Number of studies	Effect size and 95% C.I			Test of null (2-Tail)		Heterogeneity		
		Point estimate	Lower limit	Upper limit	Z-value	P-value	Q-value	df (Q)	P-value
**Parenteral**	3	0.120	0.011	1.353	-1.715	0.086			
**Combined**	1	0.030	0.000	3.298	-1.462	0.144			
**Oral**	24	0.130	0.053	0.318	-4.474	0.000			
**Overall**	**28**	**0.123**	**0.054**	**0.281**	**-4.974**	**0.000**	**0.362**	**2**	**0.834**
