# Supplementary material for: Evidence-Based Approaches for Determining Effective Target Antigens to Develop Vaccines against Post-Weaning Diarrhea Caused by Enterotoxigenic Escherichia coli in Pigs: A Systematic Review and Network Meta-Analysis
Source: Animals (Basel). 2022 Aug 19;12(16):2136. doi: 10.3390/ani12162136 (PMC9405027; doi:10.3390/ani12162136)
Supplement: Supplementary file 1 [file animals-12-02136-s001.zip › Table S3.pdf]

A. Subgroup analysis of mortality outcome by vaccine category

Groups	Number of studies	Effect size and 95% C.I			Test of null (2-Tail)		Heterogeneity		
		Point estimate	Lower limit	Upper limit	Z-value	P-value	Q-value	df (Q)	P-value
**Experimental**	12	0.303	0.182	0.504	-4.593	0.000			
**Commercial**	3	0.073	0.011	0.490	-2.697	0.007			
**Overall**	**15**	**0.275**	**0.168**	**0.451**	**-5.135**	**0.000**	**2.001**	**1**	**0.157**

B. Subgroup analysis of mortality outcome by route of administration

Groups	Number of studies	Effect size and 95% C.I			Test of null (2-tail)		Heterogeneity		
		Point estimate	Lower limit	Upper limit	Z-value	P-value	Q-value	df(Q)	P-value
**Combined**	3	0.360	0.178	0.731	-2.829	0.005			
**Intranasal**	3	0.167	0.043	0.648	-2.588	0.010			
**Oral**	8	0.199	0.071	0.563	-3.046	0.002			
**Parenteral**	1	0.086	0.002	3.704	-1.279	0.201			
**Overall**	**15**	**0.266**	**0.157**	**0.453**	**-4.879**	**0.000**	**1.806**	**3**	**0.614**
