# Supplementary material for: Evidence-Based Approaches for Determining Effective Target Antigens to Develop Vaccines against Post-Weaning Diarrhea Caused by Enterotoxigenic Escherichia coli in Pigs: A Systematic Review and Network Meta-Analysis
Source: Animals (Basel). 2022 Aug 19;12(16):2136. doi: 10.3390/ani12162136 (PMC9405027; doi:10.3390/ani12162136)
Supplement: Supplementary file 1 [file animals-12-02136-s001.zip › Table S4.pdf]

A. Subgroup analysis of the average daily weight gain outcome by vaccine category

Groups	Number of studies	Effect size and 95% C.I					Test of null (2-tail)		Heterogeneity		
		Point estimate	Standard error	Variance	Lower limit	Upper limit	Z-value	P-value	Q-value	df(Q)	P-value
**Experimental**	11	0.468	0.402	0.162	-0.320	1.257	1.165	0.244			
**Commercial**	7	1.031	0.486	0.237	0.078	1.984	2.119	0.034			
**Overall**	**18**	**0.697**	**0.310**	**0.096**	**0.089**	**1.304**	**2.248**	**0.025**	**0.794**	**1**	**0.373**

B. Subgroup analysis of the average daily weight gain outcome by vaccination route

Groups	Number of studies	Effect size and 95% C.I					Test of null (2-tail)		Heterogeneity		
		Point estimate	Standard error	Variance	Lower limit	Upper limit	Z-value	P-value	Q-value	df(Q)	P-value
**Intranasal**	3	1.519	0.749	0.561	0.051	2.988	2.028	0.043			
**Oral**	12	0.446	0.382	0.146	-0.302	1.194	1.169	0.242			
**Parenteral**	3	0.849	0.777	0.603	-0.673	2.371	1.093	0.274			
**Overall**	**18**	**0.696**	**0.312**	**0.097**	**0.086**	**1.307**	**2.236**	**0.025**	**1.674**	**2**	**0.433**
